# Probabilistic reinforcement precludes transitive inference: A preliminary study

**DOI:** 10.3389/fpsyg.2023.1111597

**Published:** 2023-03-30

**Authors:** Héctor O. Camarena, Óscar García-Leal, Julieta Delgadillo-Orozco, Erick Barrón

**Affiliations:** ^1^Centro de Estudios e Investigaciones en Comportamiento, Universidad de Guadalajara, Guadalajara, Jalisco, Mexico; ^2^Department of Environmental Sciences, University of Guadalajara, Guadalajara, Mexico; ^3^School of Doctoral Studies and Research, Universidad Europea de Madrid, Madrid, Spain; ^4^Basic Psychology Department, University of Guadalajara, Guadalajara, Mexico

**Keywords:** associative strength, transitive inference, probabilistic reinforcement, Symbolic Distance Effect, serial position effect

## Abstract

In the basic verbal task from Piaget, when a relation of the form if A > B and B > C is given, a logical inference A > C is expected. This process is called transitive inference (TI). The adapted version for animals involves the presentation of a simultaneous discrimination between stimuli pairs. In this way, when A+B−, B+C−, C+D−, D+E− is trained, a B>D preference is expected, assuming that if A>B>C>D>E, then B>D. This effect has been widely reported using several procedures and different species. In the current experiment TI was evaluated employing probabilistic reinforcement. Thus, for the positive stimuli a .7 probability was administered and for the negative stimuli a .3 probability was administered. Under this arrangement the relation A>B>C>D>E is still allowed, but TI becomes more difficult. Five pigeons (Columba Livia) were exposed to the mentioned arrangement. Only one pigeon reached the criterion in C+D− discrimination, whereas the remaining did not. Only the one who successfully solved C+D− was capable of learning TI, whereas the others were not. Additionally, it was found that correct response ratios did not predict BD performance. Consequently, probabilistic reinforcement disrupted TI, but some positional ordering was retained in the test. The results suggest that TI might be affected by associative strength but also by the positional ordering of the stimuli. The discussion addresses the two main accounts of TI: the associative account and the ordinal representation account.

## Introduction

Transitive inference (TI) can be defined as indirect learning that emerges when some form of serially ordered relationships between stimuli are trained. This kind of learning is formally represented in classical logic as if A>B and B>C, then A>C, where A > C is the untrained or “inferred” relationship. Since this kind of inference can be established only by taking the extreme elements (i.e., A and C), other procedures involving more than three stimuli have been developed in humans and animals (for a review, refer to Siemann and Delius, 1993, or Vasconcelos, 2008).

One of the most employed experimental assessments of TI involves the presentation of at least four pairs of stimuli: A + B−, B + C−, C + D−, D + E−, referred to as adjacent pairs, during a training phase, where “+” and “–” denote the presence and the absence of reinforcement after the stimulus is chosen, respectively. Subsequently, during a test phase, no-adjacent pairs (i.e., BD, AC, AD, CE, BE, AE) are presented without reinforcement or under no differential reinforcement. The preference for B over D is usually regarded as proof of TI, if such preference remains above chance levels.

Performance during adjacent and non-adjacent pairs is usually analyzed to assess two main effects: The serial position effect (SPE) and the Symbolic Distance Effect (SDE). SPE is reported as a “U” like-shape when correct responses are plotted, going from A+ to D+ during training, meaning that more extreme pairs are easier to solve than inner pairs. SDE is reported during the test when non-adjacent pairs are presented. SDE refers to an increase in the rate of correct response as symbolic distances between no-adjacent pairs increases (from BD to AE), BD being the worst solved pair.

The transitive inference was initially regarded as a consequence of some form of a language-dependent cognitive process. Piaget (1923) predicted that this form of reasoning appears in the later stages of cognitive development (e.g., after 7 years old). Nevertheless, further experiments challenged this assumption. For instance, Bryant and Trabasso (1971) showed evidence of TI in children before seven years old. Further research showed the first evidence of TI in monkeys (McGonigle and Chalmers, 1977). After those initial findings, TI has been explored in several organisms, such as humans (Gillan, 1981; Galizio et al., 2017), children (Mou et al., 2013), monkeys (Jensen et al., 2019a), crows (Lazareva et al., 2004), pigeons (Lazareva and Wasserman, 2012; Zentall et al., 2019), wasps (Tibbetts et al., 2019), and fish (Grosenick et al., 2007). More recent approaches have modeled TI in neural networks (Frank et al., 2003; Jensen et al., 2019b).

Several explanations about TI in animals have been addressed, appealing to biological mechanisms (Weiß et al., 2010), spatial representation (Roberts and Phelps, 1994), value transfer (Weaver et al., 1997), or the history of reinforcement (Couvillon and Bitterman, 1992). Both cognitive and biological approaches provide relevant evidence about the conditions that allow TI in animals. However, regarding the broadly defined concepts such as social species, memory, and spatial representation, the focus of this study is on more specific behavioral mechanisms involved in TI, specifically the history of reinforcement consequences administered during training.

The history of reinforcement can be evaluated at least in two ways: by assuming only the direct value acquired by each stimulus when reinforced during training or by calculating the composed value acquired when presented along with other stimuli. Both approaches require the estimation of the cumulative reinforcement ratios for each stimulus throughout the entire procedure. According to the former approach, preference for B over D can be predicted by performance during training, so that some form of inequality in associative strength provokes TI during the test. This general assumption has been widely explored, assessing the effects of a previously overtrained premise (usually D + E−) on testing performance. Thereby, if the associative strength of stimulus D is increased by overtraining, a preference for D over B would be expected (Lazareva et al., 2004; Lazareva and Wasserman, 2006, 2012). However, this prediction is usually not confirmed (see Lazareva et al., 2015 as an exception). The latter approach assumes that during training, the stimulus can gain or lose associative strength when they are presented in premise pairs so that TI can be explained by an acquired inequality in associative strength since B is never reinforced during A+B− but always reinforced during B + C−, and D is always reinforced during D + E−, but never reinforced during C + D−. However, B receives some additional associative value because it appears along with an always reinforced stimulus, A+, and a partially reinforced stimulus, C (never reinforced during B + C− but always reinforced during C + D−), whereas D appears along with a never reinforced stimulus E− and the partially reinforced stimulus C. Therefore, the transferred value is greater for B than for D. The assumption of value transfer has been proven on TI procedures (von Fersen et al., 1991). However, contradictory evidence has also been found when value transfer is controlled (Weaver et al., 1997).

We have previously tested the effects of overtraining in C+D− premise, finding that TI was unaffected (Camarena et al., 2018). Regarding those results, we design an alternative procedure to assess the effects of reinforcement history. Regarding the fact that overtraining does not seem to affect TI, neither administered to D + E− nor C + D−, in the present experiment, we manipulated the probability of reinforcement during training and assessed its effects on TI. Previous experiments have manipulated reinforcement probabilities (using the *p*-values of 0.5) in some critical premises as a way to control value transfer (Weaver et al., 1997), finding no effects in TI. In this experiment, using a procedure similar to the one previously described by Camarena et al. (2018), probabilistic reinforcement was introduced in each premise during training. Particularly, the probability of reinforcement when choosing the positive stimulus “+” was set to 0.7. The probability of reinforcement when choosing the negative stimulus “–” was set to 0.3. The rationale underlying this manipulation is that if TI is only dependent on reinforcement history, probabilistic reinforcement of all the premises should not affect TI. However, if a representation or discrimination between premises is required to establish TI, then probabilistic reinforcement should impair or distort the expected performance during the TI procedure since a discrimination between the *p*-values of 0.7 and 0.3 is more difficult than a discrimination between the *p*-values of 1 and 0. Consequently, it is expected that the introduction of probabilistic reinforcement does not affect TI.

## Method

### Subjects

The experiment began with 12 pigeons. Eight pigeons were experimentally naïve, whereas four had previous experience in a discrimination task. Because of a complete lack of response during auto-shaping or premise training, only the results from five pigeons are analyzed (four experimentally naïve and one with previous experience). All pigeons were maintained at ~80% of their free-feeding body weight.

Water was always available throughout the experiment in the individual home cages. All subjects were individually housed (25 cm × 25 cm × 30 cm) and exposed to a 12h:12h light/dark cycle, with lights on from 7:00 to 19:00 h.

### Apparatus

Four operant conditioning chambers (MED Associates, Inc., Model ENV-018MD). The boxes were 31 cm high, 24 cm long, and 31 cm wide. The front panel was divided into three columns. A 5.5 × 6 cm feeder opening, located 3 cm above the grid floor, gave access to the food when the hopper was activated and illuminated by a 2.8 w light. Over the feeder, placed 22 cm above the grid floor, there was a 2.54 cm diameter white cue key. The side columns were equipped with 2.54 cm diameter keys, also placed 22 cm above the grid floor. The three keys were 9 cm apart, center to center. The side keys could be illuminated in different colors. The 2.8 w house light was centrally located in the rear panel of the chamber, 27 cm above the grid floor. Each cage was located inside an isolated sound chamber (ENV-018V), equipped with a fan that circulated air and masked extraneous noises.

The experimental procedure was approved by the local Ethical Committee of the Center for Studies and Investigations in Behavior, by the University of Guadalajara committee for animal experiments, and met governmental guidelines.

### Procedure

Each session started with the illumination of the house light and the illumination of the central white key. A single peck in the center key turned it off and turned on the two side keys. The color of the side keys depended on the presented premise pair presented, so that A, B, C, D, and E were associated with red, green, blue, yellow, and cyan cues, respectively. The probability of reinforcing each key was seven out of 10 trials for the “+” stimulus (*p* = 0.7) and three out of 10 trials for the “–” stimulus (*p* = 0.3). Both probabilities were independent. They were controlled by random sampling without replacement. The position of each cue was counterbalanced across trials. Pecking one of the side keys resulted in turning off both of them, as well as the house light and the feeder activation depending on whether the trial would be reinforced or not. When the choice was reinforced, the feeder was illuminated, and 4 s of food access were allowed. Immediately, a 10 s inter-trial interval -ITI- started. When the choice was not reinforced, the side keys and the house light were turned off, and a 14 s ITI started. The following trial began with the illumination of the house light and the activation and illumination of the central white key. Only one response was required for choosing any alternative and correction trials were omitted (e. g., fixed ratio requirements for the selected alternative) as a way to keep the conditions similar to a Pavlovian procedure since in a Pavlovian procedure there are no correction trials or correct responses. All training sessions lasted until 200 trials of training were completed (or 200 trials for test phases) or 1 hour had elapsed.

Pigeons were trained in four simple overlapped item pairs, A + B−, B + C−, C + D−, and D + E−, based on the same procedure employed in Camarena et al. (2018). Hence, we used an ABCB design, where A means training, C represents overtraining, and B means test.

Training (i.e., A) was divided into four different phases. Each phase comprised 200 trials of a different type, depending on the phase. In phase 1, only pair A+B− was trained. In phase 2, the pairs A + B− and B + C− were trained in random order. In phase 3, only the pair C + D− was trained. For these three phases, at least an average of 80% of correct responses in two consecutive sessions was required to move from one phase to the next. Nevertheless, if the average correct responses during three consecutive sessions remained below 50%, the next phase was administered regardless of the low performance. This criterion was followed as a way to avoid a positive stimulus being regarded as being negative and vice versa. A similar criterion can be found in Gazes et al. (2014). Training finished after nine more sessions, during which pigeons were exposed to all the adjacent pairs: A + B−, B + C−, C + D−, and D+E− in random order. These nine sessions were intended to correct any performance deficits from previous phases. The criterion for phase 4 was calculated by averaging A+ and B+ performance and C+ and D+ performance, the sum of these two averages divided by two, was regarded as the overall performance. If the overall performance reached 80% or more during two consecutive sessions, pigeons move on to Test 1. With this arrangement, the amount of training sessions depended on the pigeons' performance until phase 4.

Tests (i.e., B) comprised 200 trials of non-adjacent pairs BD, AC, AD, CE, BE, and AE, presented in random order and under non-differential reinforcement (*p* = 0.5 for all stimuli). The first test lasted for four sessions.

Overtraining (i.e., C) comprised two more phases. During the first overtraining phase (phase 5), the pair C+D− was once again trained in 200 trials for only one session. Phase 6 was equal to phase 4 but reduced to two sessions.

Finally, the second test (i.e., B) was presented. It was equal to test 1, but it was presented only in a single session (see Table 1).

**Table 1:** Phases of the experiment.

	**Training (A)**				**Test 1 (B)**	**Overtraining (C)**		**Test 2 (B)**
	***Phase 1***	***Phase 2***	***Phase 3***	***Phase 4***		***Phase 5***	***Phase 6***	
Stimuli	A+ B−	A+ B− B+ C−	C+ D−	A+ B− B+ C− C+ D− D+ E−	B D A C A D C E B E A E	C+ D−	A+ B− B+ C− C+ D− D+ E−	B D A C A D C E B E A E
Sessions	80% of correct responses in 2 consecutive sessions or 3 consecutive sessions below 50%			9	4	1	2	1
Trials by Session	200	200	200	200	200	200	200	200

### Data analysis

For all statistical analyses, individual performance was grouped and averaged. To explore the effect of training and overtraining under probabilistic reinforcement on TI, we considered the average response between the stimuli for each pair, for the latest two training sessions (i.e., Phase 4) and overtraining (i.e., Phase 6). For test phases, we considered only the latest two sessions of Test 1 and the single session of Test 2. Additionally, we calculated the average percentage of errors in Phases 1 through 3, where an error was considered as the choice of the alternative with less probability of reinforcement for each pair. Therefore, a choice was considered correct, when the pigeons chose the alternative with the highest probability of reinforcement in each pair. The amount of errors was calculated as the mean percentage of incorrect responses for each phase. This analysis was performed as a way to explore deficits in discrimination. For test 1 and test 2 differences from chance were calculated for the crucial pair BD depending on the distribution obtained. When the distribution was normal *student t* was employed, when it was not normal the *Wilcoxon sign rank test* was employed.

## Results

As training proceeded the average amount of sessions required to reach the criterion and move from one phase to the other progressively increased. Probabilistic reinforcement, hence, made it more difficult to discriminate the stimulus with the highest probability of reinforcement. In phase 1, the average number of sessions was 2.2 ± 0.02 (mean±SEM), but it increased to 4.6 ± 1.12 in phase 2, where A+B− and B+C− stimulus pairs were randomly presented. More importantly and surprisingly, in phase 3, where only the pair C+D− was presented, only one pigeon showed 80% of correct responses in two consecutive sessions. In fact, four pigeons did not reach more than 50% of correct responses in three consecutive sessions. Thus, in phase 3, the average of correct responses was 25.3% 0 ± 6.19, remaining under chance level (*W* = 21; *p* = 0.026). A Friedman test comparing the amount of errors across phases that comprised training revealed significant differences between phases [χ^2^(2) = 8.4, *p* = 0.015], particularly between phase 1 (11.04 ± 4.15) and phase 3 (75.21 ± 8.92) (*p* = 0.022). Consequently, before presenting all adjacent pairs in phase 4, only C + D− pair was below chance.

**Figure 1:**
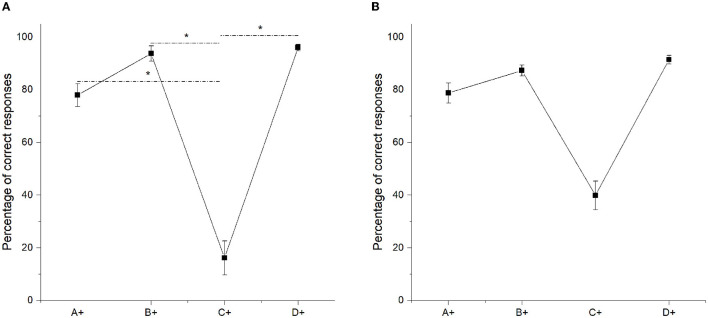
Mean percentage (SEM±) of correct responses at the end of phase 4 (eighth and ninth sessions averaged), when probabilistic reinforcement was employed **(A)** and when non-probabilistic reinforcement was employed **(B)** (Camarena et al., 2018). “*” stands for statistically significant differences.

Figure 1A shows the average percentage of correct responses in the latest two sessions of training. Each square represents the average percentage of correct responses in the pair where the stimulus + was reinforced with a probability of 0.7. After training, only in the pair C + D−, the amount of correct responses was under chance level (*W*= 22, *p* = 0.006).

The function does not resemble the U-shape idealized curve described in Vasconcelos (2008) as an example of SPE during training. However, importantly, it was similar to the function we reported in Camarena et al. (2018), using non-probabilistic reinforcement (see Figure 1B). For all the pairs, except pair C + D−, the amount of correct responses was over 80%. The main difference was that probabilistic reinforcement dropped the amount of correct responses in pair C+D− to 16.13% (see Figure 1A). Friedman's test revealed significant differences between pairs [χ^2^ (3) = 24.526, *p* < 0.001]. *Post-hoc* comparisons showed that only C+ differed from A+ (*M* = 77.94) (*p* = 0.047), from B+ (*M* = 93.72) (*p* < 0.001) and from D+ (*M* = 95.96) (*p* < 0.001). All other paired comparisons were not significant. It is worth mentioning that there were no pigeons capable of reaching the criterion in phase 4.

**Figure 2:**
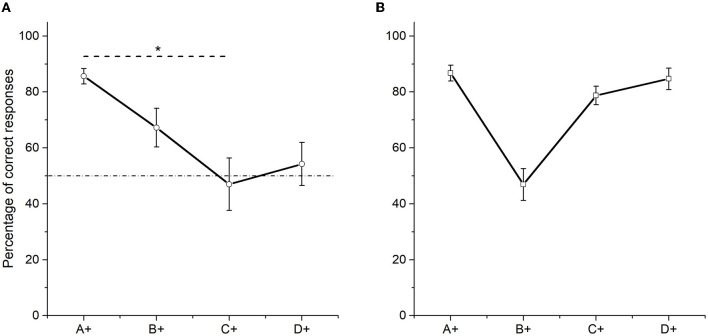
Mean percentage (SEM±) of correct responses in phase 6 (two sessions averaged), when probabilistic reinforcement was employed **(A)** and when non-probabilistic reinforcement was employed **(B)** (Camarena et al., 2018). “^*^” stands for statistically significant differences.

Overtraining increased performance in pair C+D−. Nevertheless, the percentage of correct responses in the pair C+D− did not differ from chance [*t* (9) = −0.321, *p* = 0.756]. Thus, overtraining distorted the found pattern of performance after training. It decreased correct responses in pairs B+C− (*M* = 67.21) and D+E− (*M* = 54.22) and increased correct responses in pair A+B− (*M* = 85.62). Related to Training, pair D+E− did not differed now from chance (*t* (9) = 0.547, *p* = 0.598) and only pairs A+B− (85.62) and B+C− (67.21) had a performance above chance (*p* < 0.001 and *p* = 0.035, respectively). Friedman's test comparing the amount of correct responses by premise pairs showed significant differences between premises [χ^2^ (3) = 11.64, *p* = 0.009], but only pair A + B− differed from pair C + D− (*p* = 0.003). No other differences were observed. As can be seen, the overtraining effect was different depending on the probability of reinforcement. Therefore, in this experiment and Camarena et al. (2018) experiment, the percentage of correct responses in pair C+D− increased, as well as the percentage of correct responses in pairs B + C− and D + E− decreased. However, the improvement in C + D− pair is smaller when probabilistic reinforcement is employed (see Figure 2A) than when the standard procedure is employed (see Figure 2B).

**Figure 3:**
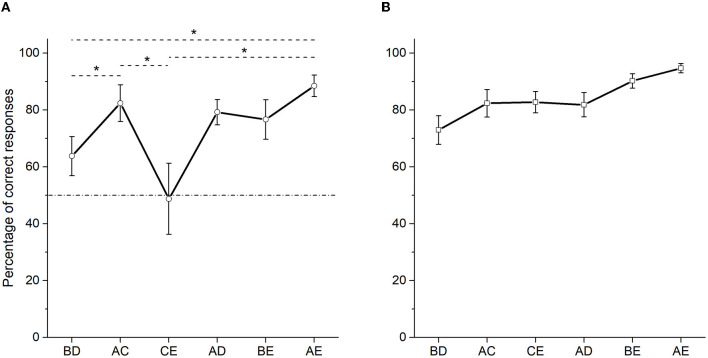
Mean percentage (SEM±) of correct responses in test 1 (third and fourth sessions averaged), when probabilistic reinforcement was employed **(A)** and when non-probabilistic reinforcement was employed **(B)** (Camarena et al., 2018). “^*^” stands for statistically significant differences.

The percentage of correct responses during test 1 (see Figure 3A) shows an increasing function related to the symbolic distance between the stimuli that form each pair. The worst performance was observed in pairs CE (*M* = 48.74) and BD (*M* = 63.75). The best performance was in the pair AE (*M* = 88.49), as expected, being the pair with the highest symbolic distance. Importantly, even when pigeons chose stimulus B more frequently during the BD pair, the percentage of correct responses (*M* = 63.75) in this pair did not differ from chance [*t* (9) = 1.994, *p* = 0.077, *d* = 2.57]. Comparisons between pairs showed statistically significant differences [χ^2^ (5) = 14.077, *p* = 0.015]. Particularly, *post-hoc* comparisons showed that BD differed from AC (*M* = 82.37) (*p* = 0.022) and AE (*p* = 0.006). CE (M = 48.74) differed from AC (*p* = 0.043) and AE (*p* = 0.012). All other pairwise comparisons were not statistically significant. Therefore, despite the observed increase in the percentage of correct responses as a function of the symbolic distance, the use of probabilistic reinforcement prevented a clear SDE, as was previously reported using the same procedure but non-probabilistic reinforcement (see Figures 3A, B). It is worth mentioning that the lowest percentage of correct responses corresponded to CE pair (48.74), which did not differ from chance [*t* (9) = −0.101, *p* = 0.922], as well as the unexpected high percentage of correct responses in the AC pair. It seems that the sequence of training made it especially difficult for the pigeons to learn about stimulus C.

**Figure 4:**
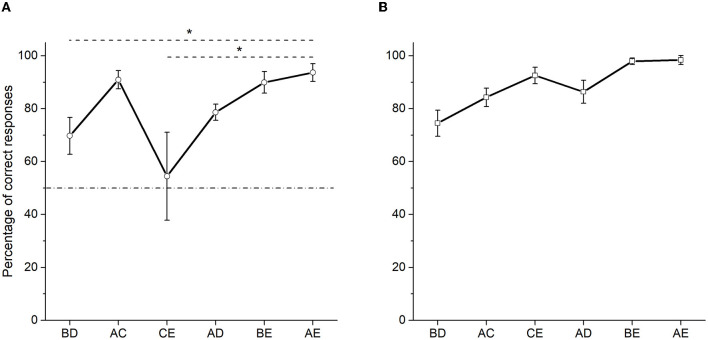
Mean percentage (SEM±) of correct responses in test 2 (two sessions averaged), when probabilistic reinforcement was employed **(A)** and when non-probabilistic reinforcement was employed **(B)** (Camarena et al., 2018). “*” stands for statistically significant differences.

Performance was quite similar comparing test 1 with test 2 (compare Figures 3A, 4A). The same result was observed using non-probabilistic reinforcement (compare Figures 3B, 4B). Like in test 1, the percentage of correct responses for the crucial pair BD (*M* = 69.72) was above 50% but did not differ from chance (*W* = 14, *p* = 0.125, *rank biserial correlation* = 0.86), in test 2 (see Figure 4A). As expected, the highest percentage of correct responses was observed in pair AE (*M* = 93.68). Once again, CE (*M* = 54.49) pair showed the lowest percentage of correct responses. For test 2, Friedman's test also showed significant differences between non-adjacent pairs [χ^2^ (5) = 14.253, *p* = 0.014], so that only the percentage of correct responses differed between pairs BD and AE (*p* = 0.011), and also between pairs CE and AE (*p* = 0.011).

Overall, these data support that the use of probabilistic reinforcement distorts the two main effects reported in TI, naming SPE and SDE. However, a pattern similar to the expected one is observed in spite of the low number of pigeons that participated in this experiment. In addition, even when pigeons chose B over D in the critical non-adjacent pair BD, the percentage of correct responses did not differ from chance. Considering these data, our results support that probabilistic reinforcement precluded TI in our experiment. With specific effects in pairs involving the worst learned stimulus C+ during training, namely AC and CE. This main effect can be partially accounted for by Pavlovian mechanisms, but the positional ordering of the stimuli also seems to be involved.

## Discussion

In this experiment, we used probabilistic reinforcement to evaluate its effect on TI, assuming that if the discrimination between stimuli is related to TI, then performance on the TI test would be affected since using probabilistic reinforcement (0.7/0.3 for stimuli + and −, respectively) is more difficult to learn than the standard procedure (see Camarena et al., 2018). Otherwise, if only the history of reinforcement is what affects TI, then test performance should not be affected since the programmed probabilities still allow the serial ordering A > B > C > D > E.

Our data support the assumption that the introduction of probabilistic reinforcement in each stimulus affects the discrimination since the beginning of the training, with an increased amount of errors from phase 1 to phase 3 and performance dropping under chance in the C + D− pair. In addition, the performance during both tests suggests that the probabilistic arrangement distorted TI. These findings can be accounted for the distortion of the serial ordering A > B > C > D > E, provoked by the lack of discrimination during C + D−. However, Pavlovian mechanisms also seem to be involved.

### Associative strength

Associative strength is referred to here in the general sense, as the capacity of a particular neutral stimulus to elicit a response from a particular organism by repeated pairings with an unconditioned stimulus. Accordingly, associative value refers to the associative strength gained by a particular stimulus. On the other hand, operant conditioning has been defined as a “Change in the frequency or probability of a response, caused by a change in the consequence or outcome of that response” (Sanabria, 2022, p. 4802). Regarding that subjects' responses do not change the programmed consequences, the employed procedure and some of the mechanisms involved are regarded as Pavlovian. However, the fact that operant contingencies can control behavior in Pavlovian procedures and vice versa (e.g., Weiss, 1972) is an issue that should not be ignored in future TI procedures (e.g., procedures where response requirements are employed).

Associative strength was evaluated in two ways; by regarding the direct value gained by each individual stimulus before the test and by regarding the history of reinforcement. Associative values were addressed by looking at correct response ratios and percentages of correct responses. Accordingly, higher percentages during training would predict a higher preference for a particular stimulus during the test, whereas equal values would predict preference at chance levels. The history of reinforcement was addressed by calculating ratios of correct responses across the entire procedure. Hence, stimulus with higher values of correct choices would be preferred during the test. Additionally, we suggested two effects in order to explain the performance in BD and CE, namely superconditioning and over expectation, as cases of stimulus compounding. Under this regard, preferences during the test could be predicted by the composed value gained by individual stimuli during training, which is presented as compounds during the test.

Assuming that subjects were responding to the direct values of each stimulus, the steadily lowered performance in C+ during Phase 4 can be explained, since C+ was below chance during phase 3. Thus, at the end of phase 4, the learned series would be A = B, B = D, and D > E, with C being the lowest one (see Figure 1). This ordering would be consistent with the BD preference at chance levels during test 1, but the CE performance at chance levels would not be consistent with the learned order.

Before test 2, the found pattern in phase 4 was distorted. Thus in phase 6, only stimulus A differed from C. On the other hand, C and D did not differ from chance, whereas A and B reached above chance performance. These differences would imply an ordering where A > C, A = B, B = C, C = D, and D = E (see Figure 2). Accordingly, during test 2, BD performance did not differ from chance. Additionally, CE again did not differ from chance. In this particular case, since B, C and D did not differ from chance in phase 6, a performance at chance levels would be expected in all pairs involving those stimuli in test 2. Nevertheless, that does not seem to be the case for pairs such as BE.

As can be seen, BD performance at chance levels seems to be predicted when B and D have equal performance with above chance levels (phase 4) and when B and D have equal performance at chance levels (phase 6). Therefore, an account based purely on the direct associative values seems inconsistent from test 1 to test 2.

In order to evaluate the reinforcement history approach, response ratios were calculated for each stimulus. Thus, by regarding the cumulate frequencies for each stimulus reinforced it should be possible to predict test performance. In this way, a stimulus with higher values will be preferred over a stimulus with lower values during the test. Following this approach, the ratio of the correct responses was calculated by dividing the absolute frequency of the S+ by the absolute frequency of the same stimulus when not reinforced (S-) (e.g., B+/B−). In the case of the initial pair, it was calculated by dividing A+ frequencies by B− frequencies. Therefore, whereas the percentage of correct responses accounts for performance after the acquisition, correct response ratios account for performance including errors during acquisition. Table 2, for each subject, values of B stimulus remained higher than values of D stimulus, before test 1. Therefore, B>D preference would be expected from correct response ratios. In the case of test 2, again all values of B are larger than values of D (see Table 3). Accordingly, the obtained BD preference in test 2 also would not be expected by correct response ratios. In regards to CE performance, correct response ratios seem inconsistent with the obtained data in tests 1 and 2. Before test 1, both C and E had the lowest values, whereas before test 2 C showed some improvement whereas E still had low values. Despite the above-mentioned differences, CE performance did not differ from chance in test 1 and test 2.

**Table 2:** Response ratios before test 1 for each subject.

	**Response ratios before test 1**				
Subject	602	603	604	701	706
A	5.63	4.58	3.78	1.93	8.64
B	4.01	2.50	3.38	2.21	3.10
C	0.17	3.96	0.89	0.01	1.32
D	0.40	0.91	0.42	0.42	1.35
**Absolute frequencies**					
A+	901	944	1002	1010	942
B−	160	206	265	523	109
B+	642	516	896	1156	338
C−	18	232	85	85	262
C+	3	918	76	1	345
D−	1046	341	983	983	254
D+	418	311	414	414	342
E−	12	132	38	38	127

**Table 3:** Response ratios before test 2 for each subject.

	**Response ratios before test 2**				
Subject	602	603	604	701	706
A	4.44	16.33	20.00	3.13	4.64
B	6.94	8.00	14.60	2.96	4.64
C	0.00	4.86	3.82	4.97	3.62
D	0.18	2.38	0.39	0.11	1.12
**Absolute frequencies**					
A+	71	98	100	72	65
B−	16	6	5	23	14
B+	111	48	73	68	65
C−	0	56	34	29	60
C+	1	272	130	144	217
D−	299	24	158	158	76
D+	53	57	62	17	85
E−	49	39	38	89	18

Regarding the above mentioned values, correct response ratios cannot account for the whole performance during test 1 and test 2. Correct response ratios could account for the improvement in C+ performance after overtraining, whereas the consistently low performance in C+ during phase 4 could be accounted for both percentages of correct responses and correct response ratios.

Stimulus compounding was addressed assuming two main effects: superconditioning and over expectation during the test. These two effects were regarded as a mere hypothesis for explaining the atypical performance found during both tests.

**Figure 5:**
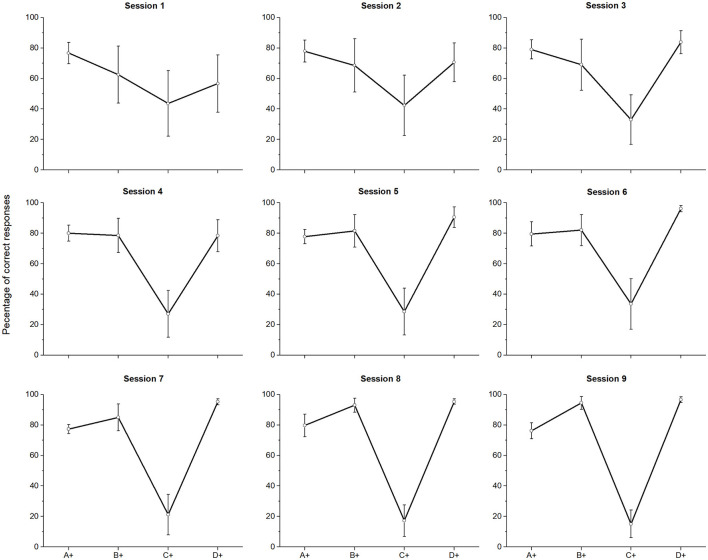
Mean percentage (SEM±) of correct responses for each session of phase 4 when probabilistic reinforcement was employed.

Assuming stimulus compounding, the poor performance for C + D− in phase 4, would have added extra associative strength to D, along with the associative strength gained during D + E−. Thus, if D was gaining associative strength during C + D−, C was losing associative strength at the same time. Thereby, performance during B + C− and C + D−, should have added very low associative strength to C, as if C was an inhibitor. Regarding the effect of superconditioning (Rescorla, 1971), a stimulus trained as an inhibitor is more difficult to condition as an excitor, whereas a compound with that inhibitor along with a new stimulus will increase the associative strength gained by the latter. Assuming an effect of superconditioning, C gained inhibitory strength during training and when C was presented along with E (always reinforced at *p* = 0.3) during the test, the compound provoked a preference that did not differ from chance, since the associative strength gained by both is so low (E was always reinforced with *p* = 0.3 and C had a probability of choice around 0.2). Therefore, superconditioning seems to account for CE performance, assuming that the test pair CE is a compound where E is a new stimulus. The obtained BD performance could be accounted for by an over expectation effect, according to this effect when two stimuli have been separately trained and have reached asymptotic performance, there will be a decrement in the conditioned response when presented together as a compound (Kremer, 1973). Assuming that B and D acquired asymptotic performance at the end of phase 4, when presented together during the test, a decrement in performance would be expected. Since BD did not differ from chance in test 1, this effect could be regarded as a consequence of over expectation. In the case of test 2, the above-mentioned effects, namely over expectation and superconditioning, seem to be absent, since in phase 6 B, C, and D did not differ from chance. Therefore, there could not be over expectation because B and D did not reach asymptotic levels and there could not be superconditioning, since C has relatively higher associative values and cannot be regarded as an inhibitor. In fact, C never reached above chance levels of performance, despite the improvement after overtraining (see Figures 5, **7**).

According to the above mentioned effects, compounding stimulus might account for BD and CE performance during test 1 but not during test 2. The fact that some form of ordering similar to SDE remained in both tests, suggests that positional ordering along with associative strength could have been used to solve the task. This suggestion is in agreement with recent studies claiming that not only associative mechanisms are involved in TI procedures (Gazes and Lazareva, 2021).

Regarding other empirical studies, several variables involved in discrimination procedures could also affect TI procedures.

### Deficits in discrimination

Response requirements, the amount of training sessions, and corrections trials seem to facilitate the learning of TI. Particularly, in cases where the discrimination between stimuli does not involve probabilities, or the probabilities involved are not overlapped between phases. In those cases, instead of an SPE during training, an ascending performance function from A+ to D+ is found, with all correct choices over chance levels (refer to, for example, Lazareva and Wasserman, 2012; Lazareva et al., 2015). Nevertheless, it is unclear whether these procedural manipulations affect TI by a Pavlovian mechanism or another mechanism. For example, response requirements involve response rates associated with particular stimuli, which in turn, could affect the preference by an increment in the attributed value. On the other hand, response requirements could increase the amount of attention toward particular stimuli by increasing their incentive salience (Williams, 1971a). Consequently, when correction trials and response requirements are introduced during TI procedures, both Pavlovian and operant contingencies can be involved. Although we sought to isolate Pavlovian contingencies by the omission of correction trials and response requirements, a purely Pavlovian account seems to be not enough for the present results (e.g., the drop in performance during C+D−), particularly, the ascending performance function during both tests, which suggests some sort of positional ordering.

There are few studies in TI that involve probabilistic reinforcement. The most direct antecedent of introducing probabilistic reinforcement in TI procedures is found in Weaver et al. (1997). However, they only employed the *p*-values of 0.5 for specific stimuli as a way to control value transfer from the S+ to S-. Specifically, they imposed that manipulation only for A and E (with premises becoming A ± B−, B + C−, C + D−, and D + E±) for Experiment 1. For Experiment 2, they employed A ± B−, B + C±, C ± D−, and D + E±. In addition, they used a criterion of 90% of correct responses between phases and an RF5 for each choice without correction trials. Therefore, the discrimination was in both experiments between the *p*-values of 1 vs. 0.5 or 0 vs. 0.5. In both experiments, the tests revealed a B>D preference over chance. Unfortunately, they did not report performance for each premise pair either during training or during the test, which does not allow for evaluating SPE, SDE, and the effects of discrimination deficits. In addition, as can be seen, their discrimination pairs were easier to solve than the procedure we employed since not all premise pairs received probabilistic reinforcement. That arrangement, along with the response requirement imposed, could have improved the discrimination between premises allowing the establishment of TI. Because of the absence of abrupt drops in performance (e.g., up to or below chance levels), the experiment from Weaver et al. (1997) does not allow a direct comparison with our results in terms of deficits in discrimination. However, based on their findings, it can be inferred that imposing probabilistic reinforcement only in a few premises neither provokes discrimination deficits nor impair TI. It is worth mentioning, that there is a recent approach from Jin et al. (2022) where all stimuli received probabilistic reinforcement, however, they employed monkeys as subjects and grouped test pairs according to symbolic distance. Therefore, our findings cannot be directly compared with theirs.

**Figure 6:**
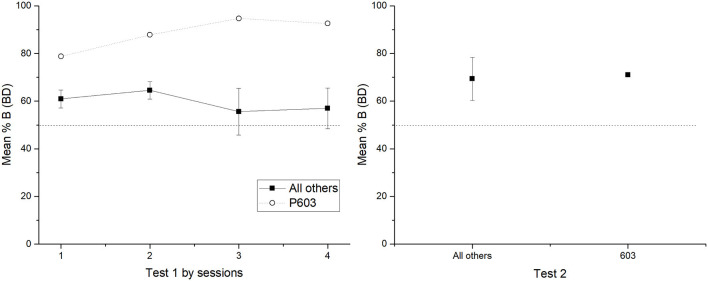
Mean percentage (SEM±) of correct responses for BD across the four test sessions of test 1 **(left)** and test 2 **(right)**. Left panel compares the grouped performance for those who did not reach the criterion in phase 3 vs the subject who reached the criterion (P603) in test 1. Right panel shows the same comparison for test 2.

The found variability in performance is not itself evidence for deficits in discrimination. However, it is worth mentioning that other studies in TI have also found such individual differences in performance where several pigeons had to be removed as training phases proceeded. For example, von Fersen et al. (1991) removed two subjects out of six that did not reach 60% of correct responses after 60 sessions. Wynne (1997) had to remove five out of eight subjects at different training phases for not reaching the expected criterion. The procedure from von Fersen et al. (1991) implied the presentation of all premises since the beginning of the training, whereas the procedure from Wynne (1997) employed a more gradual exposition of each premise. Other studies employing a gradual exposition to premises have not reported removed subjects due to deficits in performance (Lazareva et al., 2004; Lazareva and Wasserman, 2006; see Daniels et al., 2014). Roberts and Phelps (1994) have reported chance levels of response and subjects ceasing to respond when a circular arrangement is imposed. This finding can also be regarded as a consequence of deficits in discrimination when task complexity is increased. Even though the dropping rate has not been directly analyzed in TI procedures, there seems to be some relationship between the difficulty in the discrimination procedure employed and the variability between subjects (e.g., subjects responding at chance levels and subjects not reaching the criterion). The most noticeable variability between subjects we found was in phase 3, where only one subject reached the criterion (P603). Regarding the small sample size, we plotted the performance of subject P603 and the average performance of the remaining subjects during test 1 and test 2. As can be seen, subject P603 had a better BD performance during all four sessions of test 1, but during test 2 BD performance was almost the same for P603 and the remaining subjects (see Figure 6).

As can be seen in Figure 6, when removing subject 603, BD performance varies across sessions as a descending function, which suggests that BD could have reached above chance levels at least at the first two sessions. To evaluate this possibility, individual tests were run for each session, finding no differences from the chance for each session from test 1; session 1 (*M* = 60.93) [*t(3)* = 2.920, *p* = 0.061, *d* = 1.46], session 2 (*M* = 62.31) [*t(3)* = 2.788, *p* = 0.069, *d* = 1.39], session 3 (*M* = 55.58) [*t(3)* = 0.567, *p* = 0.610, *d* = 0.28], and session 4 (*M* = 56.97) [*t(3)* = 0.816, *p* = 0.474, *d* = 0.40]. Therefore, despite of the variability between subjects, the expected B > D performance seems to affect by probabilistic reinforcement.

Aside from the dropping rate and variability between subjects, the unexpectedly low performance in C + D− pair suggests a deficit in discrimination with consistent impairment on BD and CE. Previous studies have found a decline close to chance levels in performance in C + D− pair when all premises are presented (e.g., von Fersen et al., 1991; Lazareva et al., 2004). However, not all studies report whether this decline differs from chance (see Lazareva et al., 2004). Previous studies have shown that a lowering in performance in C+D− pair to chance levels does not impair TI (Camarena et al., 2018) but holds probabilities of 1 and 0 for + and – stimuli, respectively.

As it has been shown, there are several ways to add complexity during training, which can distort performance in TI procedures. Alongside, correction trials and response requirements have also shown effects during training and test that can be attributed to deficits in discrimination. For example, a dropping in C+D− has also been found using crows (Lazareva et al., 2004), with 80% of correct responses as criterion and correction trials. In this case, C+D− was near chance during training, and BD did not differ from chance (for the constant feedback group), as in our findings. Wynne (1997) also found a similar drop in performance during C+D− but using a lower criterion for correct responses (ranging from 59 to 63%) and omitting correction trials. This resemblance in performance during training and test suggests that poor discrimination during C+D− pair could have provoked the found impairment during the BD pair. Daniels et al. (2014) used pigeons in a TI procedure with six stimuli. Using only a criterion of 90% of correct responses, neither correction trials nor response requirements were employed. Additionally, they administered the complete set of premises, finding a lowering in performance to chance levels for stimuli A+ and C+. When the test was administered, preference B>D remained over chance. However, performance during CE did not differ from chance. In another study, von Fersen et al. (1991) presented all premises during 15 sessions, with correction trials, and increased the response requirement from one to eight responses for each choice. During training, C+ D− was the worst solved pair, although over chance, whereas, during the test, TI was obtained. Siemann et al. (1996) employed a procedure in which the most central premises were first trained, and the most extreme premises were trained at the end. They found an ascending function during training (plotting from A to E) that did not resemble the SPE. However, the expected TI was obtained during the test. More recent findings have found TI and SDE, without SPE, when response requirement and correction trials are imposed (Lazareva and Wasserman, 2006, 2012; Lazareva et al., 2015). Due to the fact that we omitted response requirement and correction trials (as a way to observe only the effects of Pavlovian conditioning), the mentioned findings suggest that correction trials and response requirements during choices could have reduced the task complexity in their procedures. Thereby, subjects could achieve ascending functions of performance with all pair premises over chance instead of deficits in particular premises, as we found. These findings suggest that even when probabilities are not manipulated, the absence of correction trials and response requirements can impair performance during training when performance deficits appear.

In the present experiment, the performance below chance levels in C + D− pair impaired BD and CE performance while keeping the other non-adjacent pairs above chance levels and in the ordinal expected order, where central pairs are the worst solved. In the study from Camarena et al. (2018), the performance raised above chance since the second session of C + D− training, but for subsequent sessions, C + D− never differed from chance, except when C + D− was overtrained. In the present experiment, C + D− was always below chance, reaching chance levels only after overtraining. Consequently, there seems to be a relationship between performance during C + D− and preference during the BD pair. Thereby, performance lower than chance during C + D− is associated with chance levels of preference during BD. Despite the fact that the effects of bias reversal (referred to here as overtraining) have been widely explored (Lazareva et al., 2004, 2015; Lazareva and Wasserman, 2006), the effects of deficits in performance during specific premises have not been directly addressed in TI procedures. Nevertheless, other studies have indirectly shown how to disrupt TI; for example, von Fersen et al. (1991), in their second experiment, disrupted TI by adding two new stimulus pairs, X+A- and E+F−. With this manipulation, they distorted training performance, where D+E− became the worst solved, but TI and SDE remained. However, when they added an F+X- premise in their third experiment (a case of a circular arrangement), it provoked the ceasing in response in one subject and an abruptly disrupted performance during the test, where all non-adjacent pairs did not differ from chance. A similar effect can be found in Gillan (1981) using chimpanzees, where the addition of the F+A- premise during a five-premises arrangement (from A + B− to E + F−), disrupted performance during BD, BE, and CE. Finally, the physically circular arrangement with rats from Roberts and Phelps (1994) also shows the disruption of TI. Despite the procedural differences, it seems that adding more premises and creating circular series distorts the expected BD preference and SDE by increasing the complexity of the discrimination. In the same way, the introduction of probabilistic reinforcement in a TI procedure seems to impair discrimination and distort the expected BD preference and SDE.

Regarding the above-mentioned, the present findings suggest that the introduction of probabilistic reinforcement in a TI procedure disrupts discrimination during C + D− pair, which in turn affects discrimination for the non-adjacent pairs BD and CE. The below chance levels during C + D− migth have provoked that subjects learned D− stimulus as D± (because of the D+ E− pair). Thereby, when presented along with B (also learned as B±), subjects respond at chance levels as if B ± D±. In the case of CE performance, since C was learned under chance levels during C + D− and as C− (during B + C−), a preference C < D would be expected, since if C < D and D > E, then C < E. Therefore, regarding only discrimination learning, it is unclear why CE performance did not differ from chance. This effect seems to be better addressed by an associative account.

In sum, regarding TI procedures, the statement that probabilistic reinforcement increases task complexity and impairs TI is supported by the following findings: (1) the amount of errors significantly increased as training proceeded; (2) C + D− performance remained below chance and after overtraining increased just until reaching chance levels; and (3) the crucial pair BD did not differ from chance during both tests, and (4) despite of including a criterion of 80% of correct responses between phases and nine sessions of exposure to all premise pairs, training performance remains almost the same. The third finding requires further verification since the size effect was small for test 1 but it was large for test 2. This suggests that overtraining C + D− for the second time improves BD performance and that with a larger sample size, TI could be obtained.

Overall, the present findings resemble the results previously reported by Camarena et al. (2018) but with an increased inaccurate performance during the more central pairs: C +D− during training, BD, and CE during the test (see Figures 2, 4). In addition, it is unclear why performance during both tests retained some linear order from BD to AE, with the exception of CE, which dropped to chance levels.

### Other experiments involving discrimination

**Figure 7:**
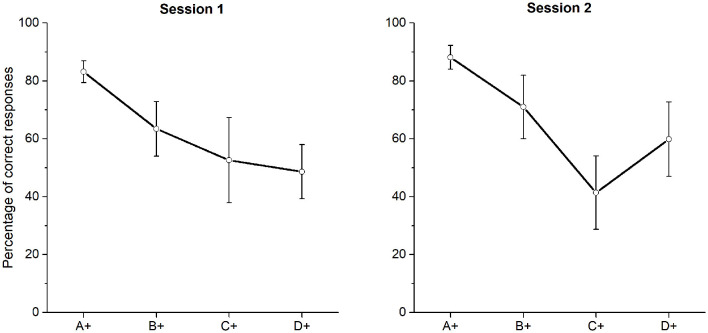
Mean percentage (SEM±) of correct responses in both sessions of phase 6, when probabilistic reinforcement was employed. Session one **(left)** and session two **(right)**.

An indirect comparison of the effects of probabilistic reinforcement can be found in the so-called ambiguous-cue problem, where a positive stimulus (P) is always reinforced, a negative stimulus (N) is never reinforced, and the ambiguous stimulus (A), which is not reinforced in the presence of P but it is reinforced in the presence of N. This could be represented as a fragment of a TI procedure (A+ B−, C±). In starlings (Vasconcelos and Monteiro, 2014), it has been shown that when the *p*-value for the stimulus P is reduced to 0.5 for one group and kept at 1 for the other group, performance is lower when *p* = *0.5* than when *p* = 1. Although the performance improved for both and reached an asymptotic level as sessions proceeded (approximately at session 12). A similar effect has been found with pigeons (García-Leal et al., 2017), where performance improved at the latest sessions but with statistically significant differences between groups. As can be seen, a discrimination between P, N, and A is easier than a discrimination between five overlapped stimuli employed here, particularly when the overlapped stimuli have reinforcement probabilities of 0.7 and 0.3. Assuming that larger differences between probabilities are more discriminable, it is possible that the lowering in performance during phase 3 was provoked by the complexity of the discrimination. We have previously shown that the introduction of the third stimulus C+ during phase 3 lowered the performance only using probabilities of 0 and 1 (Camarena et al., 2018). This lowering in performance is consistent with other discrimination procedures (e.g., Straub and Terrace, 1981; Swartz et al., 1991). However, it is not entirely clear why the C + D− performance was lowered below chance and why this low performance remained across the nine sessions of exposure to all premises during phase 4 and phase 6 (see Figures 5, 7). This consistently lower performance resembles the usual findings in the ambiguous cue problem (Vasconcelos and Monteiro, 2014; García-Leal et al., 2017) and suggests that larger exposure to C+D− could have improved performance.

Experiments in discrimination between the same and different stimuli, where probabilistic reinforcement is not involved (Cook, 2002; Cook et al., 2003) show an ascending learning curve above chance obtained with a greater or similar amount of sessions compared with TI procedures (36 sessions in Cook et al., 2003 and 100 sessions with categorical discrimination in Cook, 2002). These data suggest that a relation between the same/different is easier than ordinal discrimination between five stimuli with probabilistic reinforcement, as we employed in the TI procedure. Therefore, the introduction of probabilistic reinforcement seems to increase the difficulty of the task compared to simple discrimination procedures that do not involve overlapping stimuli. It is worth mentioning that seven out of 10 trials are reinforced in some cases, and three out of 10 are reinforced in other cases, which implies that the organism has to be exposed to at least 10 trials for learning a probability of 0.7 and other 10 trials for learning a probability of 0.3. Therefore, for establishing a performance at least over chance more trials would be required compared with a discrimination between probabilities of 1 and 0. This trend has been shown in humans, where learning the association of *p*-values such as 0.75, 0.57, 0.43, and 0.25 with particular stimuli requires at least 50 trials until reaching asymptotic levels. This learning is impaired in patients with amnesia, which suggests the involvement of memory in learning probabilities (Waltz et al., 1999). This finding would be consistent with previous research showing deficits in memory caused by brain damage impairing TI in humans (Waltz et al., 1999; Vartiani et al., 2009; Waechter et al., 2012). Consequently, the complexity of the task can overload memory resources impeding TI. Nevertheless, this assumption would not explain why C+D− performance never improved over chance, even after nine sessions of exposition to the complete sequence of premises. Therefore, in order to prove the effects of task difficulty, more evidence of humans employing a TI procedure introducing probabilistic reinforcement is required.

Along with the probabilistic reinforcement, other variables such as response requirements, deserve further consideration. Even in simpler procedures of discrimination between colors with alternating positions, it has been shown that increasing the response rate associated with each choice (e.g., FR-1, FR-5, FR-15, and FR-30) reduces the amount of errors (Williams, 1971b). The same trend has been found in “win-stay, lose-shift” procedures in pigeons, where using probabilities of 0.65 and 0.80 increases the difficulty of the task. However, the discrimination improves with FR-5 and FR-15 are introduced (Williams, 1972). Regarding the fact that we did not employ a response requirement, it seems reasonable to argue that the discrimination between premises became more difficult. Additional evidence comes from discrimination procedures, where it has been shown that pigeons require at least eight sessions of 40 trials to discriminate probabilities ranging from 0.0 to 1 (e.g., 0, 0.25. 50, 0.75, and 1) (Wasserman, 1974).

In a more recent approach, Zentall et al. (2019) using pigeons as subjects have suggested that TI can be the consequence of the tendency to *select* or *reject* stimuli. In their manipulation, they employ four different A+ stimuli as a way to reduce the tendency to reject B− stimulus during A+ B− pair and a control group with only one A+. In addition, they subdivided those groups, using colors for one group and flags for the other. They trained only one premise for each phase. With this manipulation, they found no differences between the amount of sessions required to reach the criterion (90% of correct responses) between phases. However, they found a reduced tendency to prefer B over D (due to an increased tendency to reject B); this tendency was even more reduced in the group where flags were used as stimuli. Despite this reduction in preference for B, TI remained at least in the group trained with colors. These findings suggest that complexity can be added to the task by manipulating the properties of stimuli (colors vs. flags) and the amount of stimuli. However, Zentall et al. (2019) did not find differences in the amount of sessions to reach the criterion when comparing colors and flags across phases, whereas we found differences in the amount of errors to reach the criterion. It is possible that probabilistic reinforcement and the complexity of stimuli have different effects on TI procedures.

Regarding the above mentioned findings, five main variables seem to affect the general performance during discrimination procedures and TI procedures: correction trials, order of stimuli presentation, response requirement, probability of the outcomes, and complexity of stimuli. Our findings suggest that in the absence of correction trials and response requirement, the introduction of probabilistic reinforcement impair TI, although some form of stimuli ordering is retained. The fact that stimulus A+ always had a reinforcement probability of 0.7 and stimulus E− always had a probability of 0.3 could have contributed to the found ordering by the so-called “anchoring effect.” Further studies should address the effects of probabilistic reinforcement on TI by including a control group, this would allow a direct comparison between probabilistic and non-probabilistic reinforcement.

In conclusion, using probabilistic reinforcement disrupted the expected preference B > D and C > E, but the trained stimuli were still ordered. This form of disruption in the crucial pair BD has not been previously reported employing probabilistic reinforcement. However, regarding the present results, it is not possible to state that the complexity of the task was the only variable provoking the impaired TI since some performance patterns can be explained by the direct associative strength gained by each stimulus. Super conditioning and over-expectation might be involved in those performance patterns. Consequently, both reinforcement history and the complexity of the task seem to be involved in TI procedures, even when probabilistic reinforcement is introduced. In order to disentangle the effect of both variables, some parametrical manipulations involving the five variables already mentioned are required.

## Data availability statement

The raw data supporting the conclusions of this article will be made available by the authors, without undue reservation.

## Ethics statement

The animal study was reviewed and approved by the Local Ethics Committee of the Center for Studies and Investigations in Behavior, by the University of Guadalajara Committee for Animal Experiments.

## Author contributions

HC and ÓG-L wrote the manuscript. The student JD-O ran the experiment to obtain the data. All authors contributed to the article and approved the submitted version.
